# Brazilian multicentre study of Takayasu’s arteritis in children and adolescents – preliminary results of a clinical, imaging and therapeutic study

**DOI:** 10.1186/1546-0096-9-S1-P89

**Published:** 2011-09-14

**Authors:** Maria Teresa Terreri, Gleice Clemente, Sheila K Oliveira, Marise Lessa, Clóvis Artur Silva, Adriana Sallum, Lúcia Campos, Flávio Sztajnbok, Rosana Gasparello, André Cavalcanti, Virginia Ferriani, Teresa Robazzi, Silvana Sacchetti, Maria Odete Hilário

**Affiliations:** 1 Universidade Federal de São Paulo/ Escola Paulista de Medicina, Brazil; 2 Universidade Federal do Rio de Janeiro, Brazil; 3 Universidade de São Paulo, Brazil; 4 Universidade do Estado do Rio de Janeiro, Brazil; 5 Universidade Federal de Pernambuco, Brazil; 6 Universidade Estadual Paulista, Brazil; 7 Universidade Federal da Bahia, Brazil; 8 Santa Casa de Misericórdia de São Paulo, Brazil

## Background

Takayasu’s arteritis (TA) is a chronic inflammatory disease that affects the aorta and its branches. Although it is the third most common vasculitis in childhood, reports with large number of patients are lacking.

## Aim

To evaluate the clinical features and outcome of TA in children and adolescents from Brazil.

## Methods

We retrospectively evaluated 55 patients with Takayasu’s arteritis from 8 Pediatric Rheumatology centers in Brazil. All patients fulfilled the specific classification criteria for TA that have most recently been developed for the pediatric age group. Clinical, data were collected at two time points (at disease onset and at the last available visit).

## Results

The majority of patients were girls (84%), with mean age at disease onset of 9.3 years, mean age at diagnosis of 10.6 years and a mean follow-up period of 5.6 years. The most common initial clinical manifestations included headache (56.3%), malaise (56.3%), weight loss (45.4%), dyspnea (41.8%) and limb pain (41.8%). The most common cardiovascular finding was hypertension (85.4%). The most frequent laboratory abnormality was an increased erythrocyte sedimentation rate (70.9%). The Mantoux test was positive in 38% of patients. The most common alteration was narrowing or stenosis. The most involved vessels were abdominal aorta (71.8%) followed by left (56.2%) and right renal artery (53.1%). At the last visit the most common clinical manifestations were: headache (22%), inferior limb claudication (14%) and arthralgias (12%); Cardiovascular finding included the absence or decrease of the inferior limb pulses (52%). The treatment included imunossupressors other than corticosteroids in 83.6% and anti-TNF antagonists in 7.2% of patients. Seventeen (30.9%) patients underwent surgical intervention and 5 (9%) died of complication from treatment or from disease related complications.

## Conclusion

In this large survey of children and adolescents with Takayasu’s arteritis from Brazil a delay in the diagnosis and a high rate of Mantoux test positivity were observed. A death rate similar to the described in literature was found.

